# Influence of medical didactic training on the self-efficacy and motivation of clinical teachers

**DOI:** 10.1515/iss-2023-0073

**Published:** 2024-06-21

**Authors:** Franziska Schydlo, Jasmina Sterz, Maria-Christina Stefanescu, Martina Kadmon, Sarah König, Miriam Rüsseler, Felix Walcher, Farzin Adili

**Affiliations:** Department of Anaesthesiology and Surgical Intensive Care, Klinikum Darmstadt, Darmstadt, Germany; Department of Trauma, Hand and Reconstructive Surgery, Goethe University, Frankfurt am Main, Germany; Institute for Medical Didactics and Clinical Simulation, Goethe University, Frankfurt am Main, Germany; Clinic and Policlinic for Paediatric Surgery, University Medical Center Mainz, Mainz, Germany; Faculty of Medicine, Dean’s Office, University of Augsburg, Augsburg, Germany; Faculty of Medicine, Institute for Medical Teaching and Training Research, University of Würzburg, Würzburg, Germany; University Clinic for Trauma Surgery, Magdeburg, Germany; Department of Vascular Medicine, Vascular and Endovascular Surgery, Klinikum Darmstadt, Darmstadt, Germany

**Keywords:** train the trainer, clinical teacher, didactic training, self-efficacy, motivation

## Abstract

**Objectives:**

Due to increasing workload and rising expectations for both undergraduate and speciality training in medicine, teaching in a clinical environment can be challenging. The “Train the Trainer” course, developed by CAL (Chirurgische Arbeitsgemeinschaft Lehre, Deutsche Gesellschaft für Chirurgie (DGCH)), aims to assist clinical teachers in their task. This study investigates the effect the course has on participants’ self-efficacy and teaching motivation.

**Methods:**

Prior to attending the course, participants anonymously completed a 50-question pre-course questionnaire using standardised questions to gather information on biographical data teaching experience, and validated tools measuring teaching motivation and self-efficacy (PRE). Directly after completing the course, participants evaluated it using a 25-question post-course questionnaire (POST1). At least 12 months after the course, participants received a follow-up questionnaire (POST2) by mail. This 44-question form aimed to gather biographical data, review the teaching methods participants had used since their training, and reassess their teaching motivation and self-efficacy.

**Results:**

Between June 2016 and October 2019, 20 TTT courses were held across six German medical faculties. Data were gathered from 241 participants. After the course, 182 POST2 questionnaires were mailed, 61 of which were returned (equals a 39 % return rate). The findings revealed significant increases in teacher self-efficacy (p=0.0025), identified regulation (p=0.0000), and career motivation (p=0.0044). In contrast, there was a significant decrease in introjected regulation (p=0.0048). When comparing the participants to a reference sample selected from literature, significant differences emerged in intrinsic motivation (p=0.0000) and amotivation (p=0.0025).

**Conclusions:**

Course participants already showed strong intrinsic motivation and self-efficacy before taking the course. After completing it, their confidence to meet specific teaching demands based on their abilities had increased. Notably, changes in motivational dimensions identified and introjected regulation point towards a shift in motivational sources, indicating a more self-regulated approach towards participants’ teaching activities. Further research is needed to determine how much of this change was due to course participation.

## Introduction

Clinical teachers are required to navigate a dynamic landscape of conflicting interests, caught between the demands of patient care, a shortage of skilled staff, personal career objectives, work–life balance, and the expectations of medical students and junior doctors regarding their clinical training. Moreover, the didactic methods and attitudes needed for structured knowledge and skill transfer are not usually covered in medical school or specialist training, often making them inadequately or entirely unavailable to clinical teachers. The in-depth instruction and practical execution of advanced teaching skills can help manage these competing responsibilities more effectively [1].

In 2013, the modular course **“Train the Trainer” (TTT)** was designed by CAL (Chirurgische Arbeitsgemeinschaft Lehre, Deutsche Gesellschaft für Chirurgie (DGCH)) to professionalise the medical educational skills of clinical teachers [1]. This course is intended for medical teachers across various disciplines, regardless of their prior teaching experience.

**Self-determination theory (SDT)** is a theoretical framework that explains human behaviour by focussing on human motivation and the extent to which our actions are intrinsically motivated and self-determined [2, 3]. **Self-efficacy** is defined as the confidence in one’s ability to carry out actions successfully, thereby achieving a desired outcome [4]. Within the SDT framework, **intrinsic motivation** emerges when three innate psychological needs are met: competence, autonomy, and relatedness or social integration [2, 3]. An environment in which these needs are adequately fulfilled stimulates human growth, evolution, integration, and personal well-being; motives that prompt actions driven by intrinsic motivation and form self-determined (or **self-regulated**) behaviour patterns.

The concepts of self-efficacy and motivation have significant impact on teaching and learning processes in an academic as well as a medical context. By quantifying these factors in relation to participation in the TTT course, we aim to determine how the training can influence these parameters. Several aspects of the teaching and learning dynamics are addressed below.

**Effective teaching:** Motivated teachers aspire to introduce innovative concepts and refine their teaching methods on a regular basis [5]. Teachers with high self-efficacy focus on the existing knowledge and skills of learners [6], resulting in higher commitment and more effective instruction [7, 8]. This, in turn, elicits increased effort from students, fostering their learning success and advancing their academic performance [9, 10].

**Competence:** Self-efficacy expectations are strongly linked to the fundamental need for competence. By displaying one’s capabilities or demonstrating one’s own effectiveness in achieving personal goals, thereby providing a sense of control, a strong sense of competence enhances self-efficacy [2]. In turn, high levels of self-efficacy lead to individuals’ actively seeking out situations in which to gather more evidence of their own competence, creating a positive feedback loop.

**Professional development:** Motivated teachers are eager to expand their knowledge and skills [5] and therefore often participate in continuing education programmes. This behaviour enhances their competence – both objective expertise and perceived sense of development and mastery – thereby amplifying the positive impact not only on their professional skills, but also their self-efficacy.

**Role modelling:** Teachers in any context serve as role models for their students. The attitude they demonstrate while teaching can inspire students to further develop their skills and recognise their abilities [11], increasing their own intrinsic motivation [12] and self-confidence.

**Resilience:** High levels of self-efficacy, in particular, are crucial in strengthening one’s resilience [13]. Believing in one’s capabilities to overcome challenges contributes notably to their resolution, aids in problem-solving, and increases resistance to stress and external demands amidst a field of competing interests [1, 14, 15].

**Job satisfaction:** High degrees of motivation and self-efficacy are reflected in a strong sense of job satisfaction that ideally occurs not just while teaching, but during other routine tasks [16] and, ultimately, throughout the entire professional career. It correlates with enhanced performance [16], [17], [18] and promotes personal resilience, helping to prevent stress and exhaustion in employees. Notably, job satisfaction not only greatly affects personal well-being [3, 18]; it also has significant implications on micro- and macro-economic developments as well as on society as a whole [17, 19], [20], [21].

The TTT was designed to make effective teaching more accessible and integrate it into daily clinical practice without significant resource expenditure. However, this educational strategy does not solely look towards enhancing teaching methods and advancing course participants’ academic objectives; if the above-described effects are observable, the participants will reap multiple benefits, leading to better future navigation of their challenging environment.

This study aims to explore how participation in didactic training influences the self-efficacy and teaching motivation of clinical teachers. Additional effects of the course were evaluated by asking the participants about their prior didactic experiences, didactic methods they had implemented after the course, and the frequency of their feedback.

## Methods

The TTT course runs for 7  hours distributed over 2 days, covering basic didactic principles and methods and utilising group work and simulations to demonstrate and implement them (Table 1). A significant portion of this course requires self-learning and practical application of contents. All methods taught in the course are designed to be easily integrated into clinical routine. As part of the course’s preparation and follow-up assignment, participants are asked to incorporate the methods they have learned into a course they have planned themselves.

Participants of this study were all individuals who took part in TTT courses held between June 2016 and October 2019. Their participation was voluntary, anonymous and not tied to their course results. General conditions were briefly explained before the questionnaires were handed out. Samples used in the original studies previously conducted to validate the psychometric parameters were employed as reference groups in our statistical analysis [4, 22].

**Table 1:** Content of the TTT course.

	Content	Method	Duration
Day 1			

1	Registration	Registration; PRE-questionnaires	20 min
2	Introduction, inquiry into participants’ expectations Learning objectives, agenda	Moderation cards, “body map” Flipchart presentation	30 min
3	Reflection on teaching in clinical routine	Group work, Securing results through presentation and discussion in plenum	60 min
4	Briefing – debriefing – feedback	Impulse lecture	20 min
5	Simulation of a typical teaching situation (training briefing – debriefing – feedback)	Role-playing game	3×30 min
6	Reflection on the first day of the course	One minute paper	5 min

**Day 2**			

1	Reflection on the first day of the course, agenda	Flipchart presentation	5 min
2	Constructive alignment	Impulse lecture	15 min
3	“Teachable moments”	Impulse lecture	15 min
4	Recognise and use “teachable moments”	Group work, presentation in plenum	30 min
5	Teaching and evaluation methods in everyday clinical practice: Peyton 4-step-approach, mental training, fill-ins	Group work, Simulation	3×20 min
6	Assessment methods in workplace-based training: DOPS, MiniCex Develop and test checklists	Impulse lecture Group work Simulation	15 min 15 min 15 min
7	Motivation	Impulse lecture Group work, discussion	15 min 30 min
8	Reflection on methods learned	Discussion in plenum/collecting ideas on how to transfer methods learned into one’s own lessons	10 min
9	Revision of preparation assignment – integration of new methods into the course planned at home	Individual work Presentation in small groups, structured feedback	20 min 30 min
10	Outlook on further TTT courses, feedback, written evaluation	Target Written evaluation POST1 questionnaire	15 min

Psychometric tools were employed to measure the participants’ self-efficacy across two areas. **“Generalised self-efficacy” (GSE)** gauges an individual’s confidence in their ability to address everyday challenges [4]. **“Teacher self-efficacy” (TSE)** specifically probes into teachers’ belief in their ability to meet distinct requirements of their profession [4] (Table 2).

**Table 2:** Sample items from the generalised and teacher self-efficacy scales (excerpt) according to Schweizer, Jerusalem [4, 23, 24], as well as sample items from the Physician Teaching Motivation Questionnaire (excerpt) according to Dybowski and Harendza [22].

Exemplary statement	Parameter
It is easy for me to stick to my aims and accomplish my goals.	Generalised self-efficacy
I remain calm when facing difficulties because I can rely on my coping abilities.	Generalised self-efficacy
I am convinced that I am able to successfully teach all relevant subject content to even the most difficult students.	Teacher self-efficacy
Even if I get disrupted while teaching, I am confident that I can maintain my composure and continue to teach well.	Teacher self-efficacy
During teaching, I am completely in my element.	Intrinsic motivation
I teach because I am convinced it’s a physician’s duty to pass on his knowledge.	Identified regulation
I teach because otherwise I would have a bad conscience towards my colleagues.	Introjected regulation
I teach most of the time because my supervisors expect it from me.	External regulation
I teach because I need the lessons to accomplish my occupational objectives.	Career motivation
I teach although teaching is rather irrelevant to me in comparison to my other occupational activities.	Amotivation

To determine participants’ motivation regarding their teaching activities, the **Physician Teaching Motivation Questionnaire (PTMQ)** [22] was integrated into the questionnaires. The PTMQ, built around self-determination theory (SDT), is specifically designed to measure various dimensions of clinical teachers’ motivation. It differentiates between intrinsic and extrinsic motivation, with further subdivisions for identified regulation, introjected regulation and external regulation. It also factors in career motivation and amotivation (Tables 2 and 4).

**PRE-survey:** Before the course, participants were given a 50-question anonymous PRE-questionnaire. This questionnaire enquired about their biographical information, prior teaching experience, and the regularity of their feedback to students. The survey included a variety of question types, such as multiple-choice, Likert scale, and free-text. It incorporated validated scales to measure generalised self-efficacy (GSES; 10 items, four-point Likert scale), teacher self-efficacy (TSES; nine items, four-point Likert scale) [4, 23, 24] and motivation (PTMQ; 18 items, five-point Likert scale) [22]. All questionnaires used in this study can be found in the Supplementary Material.

**POST1-survey:** After the course, participants were requested to assess it using 25 course-related anonymous questions (multiple choice, Likert scale, free text), referred to as POST1.

**POST2-survey:** Participants received a 44-question follow-up questionnaire by mail at least 12 months after completing the course. They were asked to give demographic information, details on their current didactic tasks, and their application of methods learned on the course, including the frequency of their feedback (multiple choice, Likert scale, free text). Additionally, a second assessment of generalised and teacher self-efficacy [4, 23, 24] as well as teaching motivation [22] was made using the parameters previously applied in the PRE-survey.

Participants’ responses were recorded as ratings on the Likert scale, each represented by their numerical value. Multiple-choice answers were assigned corresponding numerical codes. Free-text responses underwent analysis and grouping. Individual item values from the Likert scale resulted in scores for GSE, TSE, and motivation, which were analysed as continuous interval-scaled variables. Forms returned with incomplete answers to GSE, TSE, or PTMQ scales were excluded from further analysis. Statistical testing was performed using BiAS version 11.12 (epsilon-Verlag 2022, Goethe University Frankfurt). We would like to thank Prof. Dr. E. Herrmann of the Institute for Biostatistics, Goethe University Frankfurt, for her assistance with the statistical analysis of our data.

After collection of the questionnaires had been completed, PRE and POST2 questionnaires were matched based on participants’ biographical information while preserving anonymity. A select number of forms that could not be assigned based on these criteria was excluded from matched analysis, resulting in a sample size of 51 matched pairs of PRE and POST2 questionnaires.

GSE, TSE, and PTMQ scores were analysed for changes pre- and post-course using a paired t-test. Similarly, scores from the PRE sample were compared to those from a specified reference group for each parameter using a two-sample t-test, employing Welch’s t-test in instances of unequal variances. The original samples used to validate the psychometric parameters were used as reference groups [4, 22].

To evaluate alterations in the frequency of participants’ providing feedback before and after completing the course, the five response options (“never”, “rarely”, “occasionally”, “frequently,” and “always”) were consolidated into two categories (“frequently” and “rarely”). Subsequently, the collected data were analysed using McNemar’s test for paired samples with a sample size of n=48.

## Results

From June 2016 to October 2019, 20 TTT courses were conducted at six medical faculties in Germany. During the PRE-survey, data were collected from a total of 241 participants, 193 of which were doctors (80.1 %), 38 other medical staff (15.8 %), and nine from non-medical professional groups employed by the faculty (3.7 %). The participants’ ages varied between 22 and 58 years, with a median age of 37 years. The POST1 evaluation form was completed by 233 participants immediately after the course. A total of 182 questionnaires were distributed from 12 months (median 19 months) after course completion; the response rate was 39 % (n=61).

### Motives for taking the TTT course and previous didactic experience

Out of 193 doctors participating in the course, 92 (48 %) attended the course as part of their postdoctoral qualification, while 61 (32 %) did so out of personal interest. Thirty-seven participants (19 %) attended for both reasons.

Among all participants, 170 participants (71 %) stated that they already had previous didactic experience (Table 3). Occasions on which the participants had already taught by themselves were student placements (73 %; n=177), seminars (71 %; n=171) and lectures (n=128 or 53 %). Only 28 participants (12 %) had not previously taken part in any teaching or examination activities.

**Table 3:** Teaching activity of participants before and after taking the TTT course.

	PRE				POST2			
	Total (n=241)		Doctors (n=193)		Total (n=61)		Doctors (n=58)	
Lecture	128	53.1 %	114	59.1 %	31	50.8 %	29	50.0 %
Seminar	171	70.9 %	156	80.8 %	45	73.8 %	45	77.6 %
Placement	177	73.4 %	159	82.4 %	44	72.1 %	42	72.4 %
Licensing examination	41	17.0 %	34	17.6 %	18	29.5 %	17	29.3 %
OSCE	70	29.0 %	70	36.3 %	22	36.1 %	22	37.9 %
In-house assessment	65	27.0 %	56	30.6 %	2	3.3 %	2	3.4 %
None	28	11.6 %	11	5.7 %	2	3.3 %	2	3.4 %

### Self-efficacy

**Figure 1:**
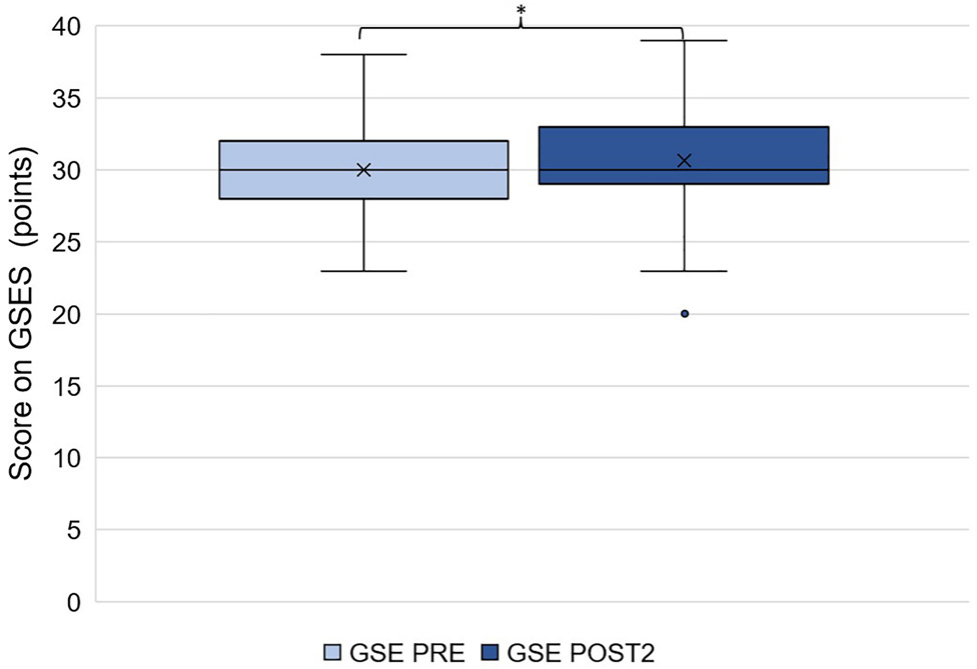
Generalised self-efficacy of TTT participants before and after taking the course (n=51, X corresponds to the mean, the box corresponds to the first to third quartile, the whiskers 5 % and 95 % quantile, individual points outliers).

**Figure 2:**
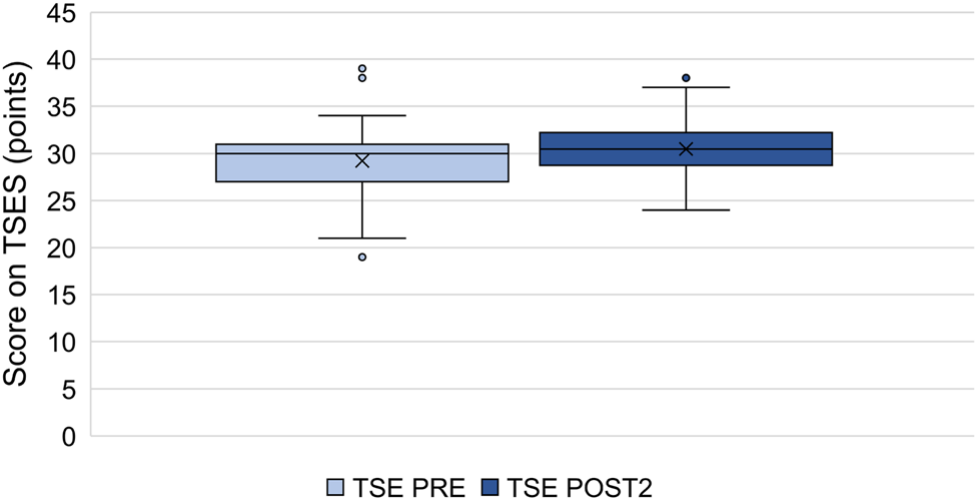
Teacher self-efficacy of TTT participants before and after taking the course (n=51, X corresponds to the mean, the box corresponds to the first to third quartile, the whiskers 5 % and 95 % quantile, individual points outliers).

The comparison of samples before and after the course did not reveal significant changes in generalised self-efficacy (n=50, p=0.1926). However, a statistically significant increase in teacher self-efficacy was observed (n=48, p=0.0025) (Figures 1 and 2).

**Figure 3:**
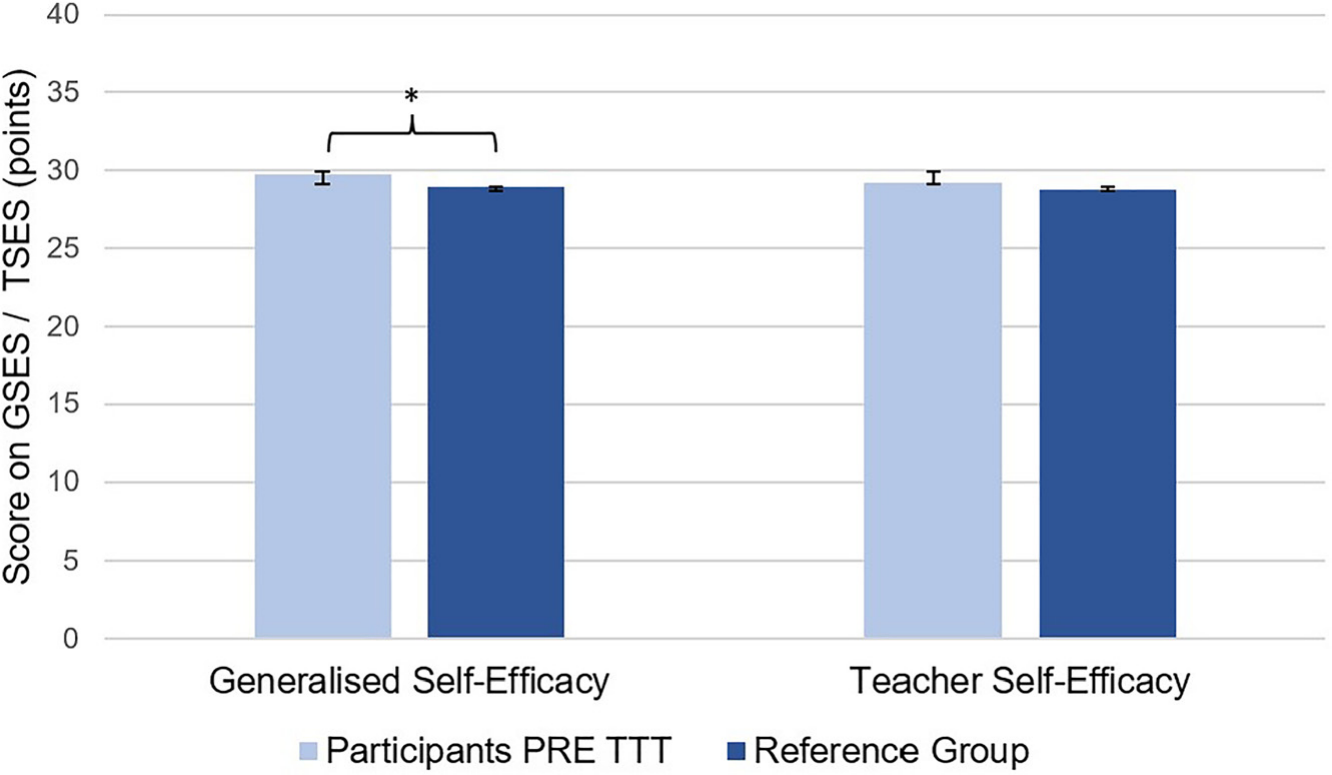
Generalised and teacher self-efficacy of participants before taking the course vs. the reference group (mean values and standard deviation).

Before taking the course, participants demonstrated a significantly higher generalised self-efficacy compared to the chosen reference group (n=236 and n=299, respectively; p=0.0085; Figure 3). There was no significant difference in teacher self-efficacy between the two groups (n=216 and n=292, respectively, p=0.1429; Figure 3).

### Teaching motivation

The comparison of samples before and after the course revealed a statistically significant increase in identified regulation (n=50, p=0.0000) and career-related motivation (n=50, p=0.0044). Meanwhile, there was a statistically significant decrease in introjected regulation (n=50, p=0.0048). No substantial differences were found for the other parameters.

**Figure 4:**
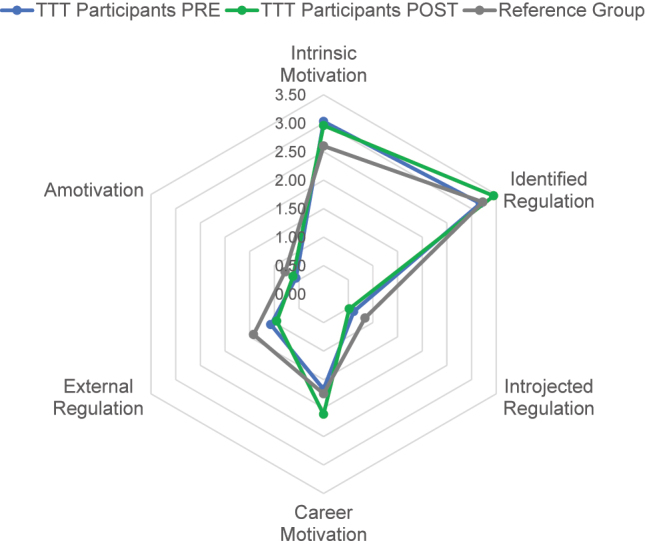
Motivation dimensions (Likert scale points) of course participants before and after completing the course as well as the reference group as seen in the original study [22].

Significant differences were observed in the parameters of intrinsic motivation and amotivation between the reference groups and course participants in the PRE-survey. Participants’ intrinsic motivation was significantly higher (n=217 and n=229, respectively; p=0.0000), and their amotivation significantly lower (n=218 and n=229, respectively; p=0.0025, Figure 4).

### Evaluation of best-rated course parts

In the POST1 evaluation, participants were asked to identify the most effective parts of the course. Role-playing “briefing–debriefing–feedback” was considered the most effective by 26 % of participants. Meanwhile, 9 % rated Peyton’s “4-step approach” highly effective, and 6 % found “teachable moments” most effective. The practical exercises in their entirety were preferred by 7 %, while 11 % found all group work to be particularly beneficial.

### Implementation of methods taught in the course

In the POST2 survey, participants were asked about the teaching methods they had applied recently, with the opportunity to provide multiple responses. Of the 61 participants, 79 % reported using the “debriefing/feedback” method, followed by the closely related “briefing” method, used by 70 % of the participants. The third most frequently used method was “teachable moments”, reported by 46 %. The participants had used other available methods such as “MiniCex,” “4-step approach,” “mental training,” and “DOPS” considerably less often.

### Feedback

In the PRE and POST2 surveys, participants were asked to estimate the frequency of their feedback to students. After matching PRE and POST2 samples, excluding incomplete questionnaires, data from 48 participants were analysed. There were no statistically significant changes in the frequency of feedback (p=0.1185).

## Discussion

In the field of teaching, self-efficacy and motivation are central concepts, and various examples for their application can be found in literature. However, the majority of such studies are conducted in the area of primary and secondary education; studies referring to higher education mostly focus on students, and those set in a clinical context usually examine the doctor–patient dynamic. This study specifically concentrates on teachers in a clinical setting.

As the TTT course is held at university hospitals, the majority of its participants, and thereby our study sample, are situated in a clinical context amongst a multitude of competing demands (as mentioned above). Seeing as teaching in such an environment is especially challenging, the potential benefits offered by didactic interventions, specifically the TTT, are immense. Investigating its impact – a first for this course – provides a valuable foundation not only for designing and refining didactic courses in a medical context but also for future studies in this expanding field of research.

### Methodological competence – effectiveness of course content and implementation of learned methods

The primary objectives of the TTT course revolve around imparting methodological skills [1]. Participants gain insights into didactic theories and principles, learning techniques such as conducting briefings and debriefings, providing feedback, identifying and utilising teachable moments, implementing the 4-step approach for specific actions, and planning their own lessons. The immediate applicability of the content aims to allow for its effortless and efficient use in everyday clinical practice. Investigating how participants applied the course content post-practice was therefore crucial to this study.

The majority of participants rated the role play on briefing, debriefing, and feedback positively, naming it the most effective part of the course. These same topics also most frequently appear amongst the teaching methods that have been implemented in clinical routine. A possible reason for this could be that a significant amount of time is devoted to this role-play during the course, allowing participants to go through the process repeatedly, akin to a learning spiral. Additionally, the first-hand experience gained from the simulation of a teaching situation leaves a lasting impression on the participants (for more details, see below).

Feedback is crucial in the educational process for several reasons. Constructive feedback enhances learners’ confidence and promotes self-reflection on their performances, helping them understand the reasons behind their successes or failures [18]. Recognising an internal locus of causality strengthens learners’ self-efficacy and helps them develop a realistic perception of their own strengths and weaknesses. Similarly, instructors also benefit from constructive feedback about their teaching methods, which enhances their sense of competence and positively impacts their intrinsic motivation to teach [18].

The course exercises are designed to help participants articulate effective feedback so that the feedback’s supportive function is highlighted and ensures the recipient accurately interprets and implements it. As mentioned above, participants have rated the role play on briefing, debriefing, and feedback as the most valuable part of the course, suggesting that the importance of its contents has been successfully communicated. Changes seen in the frequency of participants giving feedback support this conclusion as well; however, they were not statistically significant.

The frequent listing of the “teachable moments” exercise as a valuable part of the course implies participants prefer spontaneous teaching methods to those that require extensive preparation.

Notably, the presentation on “constructive alignment” and the accompanying checklist design exercises are hardly mentioned in the participants’ lists of the course’s most effective contents. “Constructive alignment” refers to the drafting of a clear, competency-based learning objective, followed by the selection of teaching and assessment tactics to efficiently relay the learning material and evaluate whether it has been successfully imparted. This process, while often overlooked, is crucial for effective teaching and learning. Only by matching the content’s form to a specific assessment method, students are placed in a suitable position to develop the required skills [25]. To emphasise this connection’s importance for effective instruction, this course component has been expanded in later TTT courses to include the development of Specific, Measurable, Achievable, Relevant, and Time-bound (SMART) learning objectives and the selection of appropriate assessment methods.

### Self-competence – self-efficacy and self-regulation

The TTT course not only imparts methodological skills but also aims to promote self-competence [1]. This involves increasing the participants’ confidence in their own abilities, as marked by self-efficacy, and the enhancement of their motivation to teach. The participants’ intrinsic motivation, especially their drive to engage in teaching, and ways of influencing it, is of particular interest to this study. Therefore, the course methodology has been specifically designed to encourage autonomy and active participation. By stimulating a high-level of self-regulated behaviour and engineering situations in which participants experience their own competence, the course aims to strengthen their intrinsic motivation, thereby ensuring their future dedication to teaching [18]. For this reason, measuring self-efficacy and motivation, particularly probing into external and internal sources of regulation, and interpreting them within the framework of self-determination theory were another essential objective of this study.

### Factors influencing self-efficacy and their use in the TTT course

Several factors involved in an intervention can impact an individual’s self-efficacy positively or negatively, thereby altering their behaviour [26]. Here, we will discuss three of these factors: the successful completion of an activity or the achievement of a goal that previously seemed unattainable vs. personal failure at it, experiencing the above through an observed proxy, and the experience and cognitive classification of an emotional or physiological state of arousal.

By partaking in an interactive role-play exercise, such as the briefing–debriefing–feedback session, participants can directly apply a learned method, encountering an immediate sense of achievement, regardless of any previous teaching experiences. The repeated performance of such exercises, establishing a pattern of repeated success, can improve an individual’s self-evaluation of competence and enhance their confidence in the method. When roles are alternated, it provides an opportunity for participants to observe others during their experiences. The participant’s individual cognitive appraisal of both experiences becomes more positive when fewer external factors simplify task performance, prior teaching experience and other characteristics vary within the group of participants successfully fulfilling the set task, and it becomes obvious that not all tasks are easy for all participants. The potential onset of nervousness or fear when facing an assignment is correlated to a lower expectation of self-efficacy. However, perceiving the role-playing situation as non-threatening encourages a proactive approach towards the task, resulting in long-term reduction of fear and anxiety, improvement in performance, and, ultimately, an increase in self-efficacy.

Across different areas of life in which one’s competence can be perceived, the generalisation of a specific experience may take place, possibly resulting in a change of generalised self-efficacy [27]. However, this effect is most likely to occur in activities that are similar to those in which higher self-efficacy has been achieved before [28].

Therefore, experiences gained in the TTT course could potentially alter the participants’ generalised self-efficacy; at the same time, gaining skills on the job (particularly during speciality training) or even personal experiences may also affect self-efficacy, acting as confounding factors. To accurately assess this effect, we employed measurement tools aimed at both generalised and teacher self-efficacy (GSES and TSES, respectively) in our surveys. The results showed that while the initially high levels of GSE remained almost unchanged post-course, there was a significant increase in TSE. This suggests that the skills acquired during the course improved self-efficacy for related tasks – in this case, teaching. However, this effect was too specific to influence generalised self-efficacy.

### Teaching motivation and the process of internalisation among TTT participants

Self-Determination Theory (SDT) suggests that motives for action originate from a continuous spectrum including amotivation, extrinsic, and intrinsic motivation, revealing an increasing degree of self-regulation and autonomy, rather than resorting to a simple dichotomy between extrinsic and intrinsic motivation (Table 4). Within SDT, the term “internalisation” refers to a process during which behaviours initially regulated externally become more voluntary, i.e. are being driven by intrinsic motivation [2, 22].

**Table 4:** Motivation spectrum according to SDT [2]; also refer to Table 2 [22].

Amotivation	Extrinsic motivation				Intrinsic motivation
Lack of regulation	External regulation	Introjected regulation	Identified regulation	Integrated regulation	Intrinsic regulation
Lack of intention	External expectations, external incentives or punishment	Self-control, internal incentives or punishment (e.g., feelings of pride or guilt)	Conscious recognition of personal meaning	Conscious implementation in congruence with one’s own values	Interest, joy, satisfaction

→ Increasing self-regulation, autonomy →					

Contrary to our expectations, the results of this study indicate that the course did not significantly alter the participants’ intrinsic motivation to teach. However, their intrinsic motivation before taking the course was significantly higher than that of the chosen reference group (which was comprised of clinical teachers in a university hospital setting, the study being unconnected to a specific didactic intervention [22]). This outcome could be due to selection bias; it might also indicate the participants’ motivation to teach was so high already that being involved in the course no longer made any substantial difference.

Further analysing the results regarding the other dimensions of motivation, the statistical changes that have been found can be interpreted as a shift of motivational sources towards a more self-regulated behaviour, despite the fact that no changes were found in intrinsic motivation itself. A significant decrease in introjected regulation after the course suggests fewer participants are now teaching due to internal pressure - a result of the internalisation of previously external incentives. At the same time, the significant rise in identified regulation implies that their personal convictions are now increasingly driving participants to teach, as they are beginning to identify with their role and the accompanying values of a clinical teacher. The observed shift into a more autonomous regulatory style increases the likelihood of participants’ future commitment in teaching activities [3, 17].

The classification of career motivation (as in “incentive to be involved in teaching”) is ambiguous. Because teaching often serves as a tool for personal academic advancement, it is appropriate to regard career motivation as a subtype of external regulation. However, motivations for professional progression (“career motivation” in a broader sense) are multifaceted and include both extrinsic (e.g., income and social status) and intrinsic (e.g., knowledge expansion and personal challenge) elements, making the differentiation somewhat challenging [22, 29]. To accurately determine an internalisation process, “career motivation” needs to be thoroughly examined in subsequent studies, distinguishing between intrinsic and extrinsic regulatory mechanisms. Even so, the substantial rise in the “career motivation” factor recorded among participants can certainly be interpreted as an additional motivational resource to help persist in teaching after course completion.

## Limitations

The study’s primary constraint involves confounders affecting participant results, particularly in case of extended periods between course participation and POST2 survey. Psychometric parameters such as self-efficacy and teaching motivation in particular are influenced not only by course participation but also by further acquisition of skills in both a professional and private context. To mitigate these influences, future studies should adopt prospective controlled trials.

The study sample consists of participants in an educational training course, creating a selection bias regarding teaching motivation. Doctors not involved in teaching or not taking part in didactic training would likely show different baseline values for teaching motivation. The inclusion of a control group could again have provided an advantageous balance. There is also a potential observation bias in the survey due to the nature of the study’s inquiry. Despite this unavoidable bias, efforts to minimise it were made by ensuring the survey’s anonymity.

Dybowski et al.’s [8] study conceived the Physician Teaching Self-Efficacy Questionnaire (PTSQ), which was designed specifically for clinical teachers, thus more precisely reflecting the “teacher self-efficacy” parameter. Although it was not employed for the ongoing study due to comparability issues, it is recommended for future research.

## Conclusions

The concepts of self-efficacy and self-determination theory are well-established in education, sociology, and psychology. Significant research has been conducted on the influence of self-efficacy among schoolteachers, students and patients. However, teachers in a clinical setting remain an overlooked research group, with limited studies dedicated to their self-efficacy and teaching motivation [8, 30, 31]. Given the potential benefits, particularly in the restructuring of medical undergraduate and speciality training [32–34], it is essential to expand research in this area.

The “Train the Trainer” course holds potential not just for skill acquisition but also for strengthening participants’ self-efficacy and motivation to teach. This can be observed through its graduates’ increased identification with the role of a clinical teacher and growing confidence in handling associated challenges. Furthermore, while intrinsic motivation remained almost unchanged, a noticeable internalisation and integration of the imparted values was shown. The results’ validity is somewhat limited due to variable factors in the duration between course completion and follow-up survey, which may have affected self-efficacy and motivation. Future controlled studies are needed to quantify this effect.

In the ongoing and future development of the TTT and similar formats, focus should be given to the hands-on exercises of the course that potentially have a positive impact on self-efficacy [30]. These segments were highly favoured by participants and considered the most effective. Nonetheless, theoretical elements of the course should not be neglected as they support the process of internalisation and serve as food for thought. In addition, the current version of the TTT emphasises the application of “constructive alignment” by including the development of SMART learning objectives and the selection of suitable assessment methods.

## Supplementary Material

Supplementary Material
