# Supplementary material for: Influence of medical didactic training on the self-efficacy and motivation of clinical teachers
Source: Innov Surg Sci. 2024 Jun 21;9(2):99–108. doi: 10.1515/iss-2023-0073 (PMC11294303; doi:10.1515/iss-2023-0073)
Supplement: Supplementary file 1 — Supplementary Material [file j_iss-2023-0073_suppl_001.docx]

**FRAGEBOGEN**

1. **ALLGEMEINER TEIL**
2. **EINGANGSFRAGEBOGEN**
3. **EVALUATIONSFRAGEBOGEN**
4. **ABSCHLUSSFRAGEBOGEN**
5. **ALLGEMEINER TEIL**

**Alter:**

**Fachdisziplin:**

**Dienstjahre:**

**Woher haben Sie von dem Kurs erfahren?**

**Warum nehmen Sie an dem Kurs teil?**

1. **EINGANGSFRAGEBOGEN (PRÄ)**
2. Welche Position üben Sie in Ihrer Institution aus?

🞎 Chefarzt

🞎 Oberarzt

🞎 Assistenzarzt

🞎 Sonstiges

1. Über welche didaktische Qualifikation/Vorerfahrungen verfügen Sie?

🞎 Master of Medical Education

🞎 Didaktikkurs im Rahmen der Habilitation

🞎 Dozenten- oder Prüfertraining

🞎 Sonstige .....................................................................................................

🞎 Keine

1. Bei welchen Lehrveranstaltungen/Prüfungen haben Sie bereits als Dozent/Tutor mitgewirkt? (Mehrfachantworten möglich)

🞎 Vorlesung

🞎 Seminar

🞎 Praktikum

🞎 Staatsexamen

🞎 OSCE

🞎 Testate

🞎 Keine

1. Fühlen Sie sich didaktisch ausreichend geschult für die Lehrveranstaltungen, an denen Sie mitwirken?

🞎 Ja

🞎 Nein

🞎 Geht so

1. Wie gerne arbeiten Sie mit Studierenden?

🞎 Sehr gerne

🞎 Gerne

🞎 Keine Meinung

🞎 Ungerne

🞎 Extrem ungerne

1. Werden Sie früh genug darüber informiert, dass Sie Studierende unterrichten sollen?

🞎 Nie

🞎 Selten

🞎 Gelegentlich

🞎 Häufig

🞎 Immer

1. Welche Aufgaben haben Sie bereits an Ihre Studierenden, nach Einweisung, zur routinemäßigen, selbstständigen Durchführung delegiert? (Mehrfachantworten möglich)

🞎 Blutentnahme

🞎 Bluttransfusion (Bedside Test + Anhängen EK)

🞎 Intravenösen Zugang legen

🞎 Infusionen anhängen

🞎 Anamneseerhebung, Aufnahmegespräch führen

🞎 Körperliche Untersuchung, Aufnahmeuntersuchung durchführen

🞎 Arztbriefschreibung

🞎 Kommunikation (schriftlich, mündlich, persönlich) mit Zuweisern

🞎 Anforderung von Konsilen und/oder Untersuchungen

🞎 Verbandwechsel (komplex; inkl. Débridement, VAC-Verband etc.)

🞎 Pleura-, Aszitespunktion

🞎 Arterielle Punktion, BGA, Arterielle Druckmessung

🞎 Magensonde legen

🞎 Blasenkatheter legen (Einmalkatheter, Dauerkatheter)

🞎 Aufklärungsgespräche für kleinere ambulante Standardeingriffe oder stationäre Diagnostik durchführen

🞎 EKG schreiben

🞎 Analgetika, Antiemetika indizieren und nach Rücksprache verabreichen

🞎 Führen einer Visite

🞎 Port anpunktieren und befahren

🞎 Angehörigengespräche führen

🞎 Codieren

1. Überprüfen Sie die korrekte Durchführung der delegierten Aufgaben und geben Sie Feedback?

🞎 Nie

🞎 Selten

🞎 Gelegentlich

🞎 Häufig

🞎 Immer

1. Wie belastet die Ausbildung von Studierenden Ihren klinischen Alltag?

🞎 Gar nicht

🞎 Wenig

🞎 Moderat

🞎 Sehr

🞎 Extrem

1. Wieviel Zeit beansprucht die studentische Lehre in Ihrem klinischen Alltag?

🞎 0%

🞎 <10%

🞎 <20%

🞎 <30%

🞎 >30%

1. Welche Tätigkeiten haben Studierende unter Ihrer Anleitung/Supervision im Operationssaal durchgeführt? (Mehrfachnennungen möglich)

🞎 Team Time Out (OP-Checkliste)

🞎 Lagerung

🞎 Abwaschen

🞎 Abdecken

🞎 Zweite OP-Assistenz

🞎 Erste OP-Assistenz

🞎 Kleinere Eingriffe (z.B. Metallentfernung, Minor-Amputation, Abszessspaltung Wundversorgung)

🞎 Knoten

🞎 Subkutannaht

🞎 Hautnaht

🞎 OP-Dokumentation (Ausfüllen eines Protokollbogens mit postoperativen Anweisungen)

1. Wo liegen Ihrer Meinung nach die Haupthindernisse für eine effektive Lehre im klinischen Alltag? (max. 5 Nennungen)

🞎 Zeitmangel

🞎 Personalmangel

🞎 Zu viele Studierende gleichzeitig vor Ort

🞎 Gestiegene Ansprüche der Studierenden an die Qualität der Lehre (Generation Y)

🞎 Mangelnde Vorkenntnisse der Studierenden

🞎 Mangelnde didaktische Kenntnisse, Fertigkeiten und Fähigkeiten der Lehrenden

🞎 Mangelnde intrinsische Motiviation der Lehrenden

🞎 Mangelnde Wertschätzung der Lehre (fehlende Anreize für Lehrende)

🞎 Gestiegene Ansprüche an Patientensicherheit, Sorge um Behandlungsfehler

Spezialisierung, Zentrumsbildung, enges Behandlungsspektrum

🞎 Mangelnde Ausbildung, fachliche Kenntnisse, Fertigkeiten, Fähigkeiten der Lehrenden

🞎 Interprofessionelle Spannungen (z.B. Ärzte-Pflege) durch lehrbedingte „Störungen“ der Stationsroutine

🞎 Diversity (mangelnde Sprachkenntnisse, soziokulturelle Unterschiede) bei Lehrenden und Studierenden

🞎 Unzureichende Lehrinfrastruktur (Zugang zu Literatur, Skills Lab, Unterrichtsräume, Lehrmaterialien)

1. Kennen Sie den Lernzielkatalog Ihres Fachgebietes?

🞎 Nein

🞎 Wenig

🞎 Geht so

🞎 Gut

🞎 Ja

| 1. Wenn sich Widerstände auftun, finde ich Mittel und Wege, mich durchzusetzen. | Stimmt nicht | Stimmt kaum | Stimmt eher | Stimmt genau |
| --- | --- | --- | --- | --- |
| 1. Die Lösung schwieriger Probleme gelingt mir immer, wenn ich mich darum bemühe. | Stimmt nicht | Stimmt kaum | Stimmt eher | Stimmt genau |
| 1. In unerwarteten Situationen weiß ich immer, wie ich mich verhalten soll. | Stimmt nicht | Stimmt kaum | Stimmt eher | Stimmt genau |
| 1. Es bereitet mir keine Schwierigkeiten, meine Absichten und Ziele zu verwirklichen. | Stimmt nicht | Stimmt kaum | Stimmt eher | Stimmt genau |
| 1. Auch bei überraschenden Ereignissen glaube ich, dass ich gut mit ihnen zurechtkommen kann. | Stimmt nicht | Stimmt kaum | Stimmt eher | Stimmt genau |
| 1. Schwierigkeiten sehe ich gelassen entgegen, weil ich meinen Fähigkeiten immer vertrauen kann. | Stimmt nicht | Stimmt kaum | Stimmt eher | Stimmt genau |
| 1. Was auch immer passiert, ich werde schon klarkommen. | Stimmt nicht | Stimmt kaum | Stimmt eher | Stimmt genau |
| 1. Für jedes Problem kann ich eine Lösung finden. | Stimmt nicht | Stimmt kaum | Stimmt eher | Stimmt genau |
| 1. Wenn eine neue Sache auf mich zukommt, weiß ich, wie ich damit umgehen kann. | Stimmt nicht | Stimmt kaum | Stimmt eher | Stimmt genau |
| 1. Wenn ein Problem auftaucht, kann ich es aus eigener Kraft meistern. | Stimmt nicht | Stimmt kaum | Stimmt eher | Stimmt genau |

| 1. Ich lehre, weil ich es sehr wichtig finde, meinen Teil dazu beizutragen, dass die Studierenden später gute Ärzte werden. | Stimmt nicht | Stimmt kaum | Keine Meinung | Stimmt eher | Stimmt genau |
| --- | --- | --- | --- | --- | --- |
| 1. Ich lehre, weil ich überzeugt bin, dass es zu den Pflichten eines Arztes gehört, sein Wissen weiterzugeben. | Stimmt nicht | Stimmt kaum | Keine Meinung | Stimmt eher | Stimmt genau |
| 1. Ich lehre vor allem, weil meine Vorgesetzten es von mir erwarten. | Stimmt nicht | Stimmt kaum | Keine Meinung | Stimmt eher | Stimmt genau |
| 1. Ich lehre, obwohl die Lehre mir eher gleichgültig im Vergleich zu meinen anderen beruflichen Tätigkeiten ist. | Stimmt nicht | Stimmt kaum | Keine Meinung | Stimmt eher | Stimmt genau |
| 1. Ich lehre vor allem, weil es zu meinen Arbeitsaufgaben gehört. | Stimmt nicht | Stimmt kaum | Keine Meinung | Stimmt eher | Stimmt genau |
| 1. Ich freue mich meistens auf die nächste Lehreinheit. | Stimmt nicht | Stimmt kaum | Keine Meinung | Stimmt eher | Stimmt genau |
| 1. Ich lehre, obwohl ich fast nie Lust darauf habe. | Stimmt nicht | Stimmt kaum | Keine Meinung | Stimmt eher | Stimmt genau |
| 1. Mein Unterricht macht mir meistens Spaß. | Stimmt nicht | Stimmt kaum | Keine Meinung | Stimmt eher | Stimmt genau |
| 1. Ich lehre, obwohl ich die Lehre oft als lästige Pflicht empfinde. | Stimmt nicht | Stimmt kaum | Keine Meinung | Stimmt eher | Stimmt genau |
| 1. Ich lehre, weil ich sonst ein schlechtes Gefühl meinen Kollegen gegenüber hätte. | Stimmt nicht | Stimmt kaum | Keine Meinung | Stimmt eher | Stimmt genau |
|  |  |  |  |  |  |
| 1. Während des Unterrichts fühle ich mich ganz in meinem Element. | Stimmt nicht | Stimmt kaum | Keine Meinung | Stimmt eher | Stimmt genau |
| 1. Ich lehre, weil ich den Unterricht benötige, um meine beruflichen Ziele zu erreichen. | Stimmt nicht | Stimmt kaum | Keine Meinung | Stimmt eher | Stimmt genau |
| 1. Ich lehre, weil ich sonst ein schlechtes Gewissen gegenüber meinen Vorgesetzten hätte. | Stimmt nicht | Stimmt kaum | Keine Meinung | Stimmt eher | Stimmt genau |
| 1. Die Lehre stellt eine Bereicherung meines Arbeitsalltags dar. | Stimmt nicht | Stimmt kaum | Keine Meinung | Stimmt eher | Stimmt genau |
| 1. Ich lehre, weil es mich beruflich voranbringt. | Stimmt nicht | Stimmt kaum | Keine Meinung | Stimmt eher | Stimmt genau |
| 1. Ich lehre vor allem, weil ich sonst Schwierigkeiten mit meinen Vorgesetzten bekäme. | Stimmt nicht | Stimmt kaum | Keine Meinung | Stimmt eher | Stimmt genau |
| 1. Ich lehre, weil mich dies in meiner Karriere voranbringen könnte. | Stimmt nicht | Stimmt kaum | Keine Meinung | Stimmt eher | Stimmt genau |
| 1. Ich lehre, weil ich die Inhalte meines Unterrichts wichtig finde. | Stimmt nicht | Stimmt kaum | Keine Meinung | Stimmt eher | Stimmt genau |

| 1. Ich bin sicher, dass ich auch mit problematischen Studierenden in guten Kontakt kommen kann. | Stimmt nicht | Stimmt kaum | Stimmt eher | Stimmt genau |
| --- | --- | --- | --- | --- |
| 1. Ich weiß, dass ich es schaffe, selbst den problematischsten Studierenden den prüfungsrelevanten Stoff zu vermitteln. | Stimmt nicht | Stimmt kaum | Stimmt eher | Stimmt genau |
| 1. Ich bin mir sicher, dass ich mich in Zukunft auf individuelle Probleme der Studierenden besser einstellen kann. | Stimmt nicht | Stimmt kaum | Stimmt eher | Stimmt genau |
| 1. Selbst wenn mein Unterricht gestört wird, bin ich mir sicher, die notwendige Souveränität bewahren zu können. | Stimmt nicht | Stimmt kaum | Stimmt eher | Stimmt genau |
| 1. Selbst wenn es mir mal nicht so gut geht, kann ich doch im Unterricht immer noch gut auf die Studierenden eingehen. | Stimmt nicht | Stimmt kaum | Stimmt eher | Stimmt genau |
| 1. Auch wenn ich mich noch so sehr für die Entwicklung meiner Studierenden engagiere, weiß ich, dass ich nicht viel erreichen kann. | Stimmt nicht | Stimmt kaum | Stimmt eher | Stimmt genau |
| 1. Ich bin mir sicher, dass ich kreative Ideen entwickeln kann, mit denen ich ungünstige Unterrichtsstrukturen verändere. | Stimmt nicht | Stimmt kaum | Stimmt eher | Stimmt genau |
| 1. Ich traue mir zu, die Studierenden für neue Projekte zu begeistern. | Stimmt nicht | Stimmt kaum | Stimmt eher | Stimmt genau |
| 1. Ich kann innovative Verändungen auch gegenüber skeptischen Kollegen durchsetzen. | Stimmt nicht | Stimmt kaum | Stimmt eher | Stimmt genau |

1. **EVALUATIONSFRAGEBOGEN (POST 1)**
2. Wie war das Verhältnis zwischen Frontalvortrag und selbstgesteuertem Lernen? (Mehrfachnennung möglich)

🞎 Zu viel Frontal

🞎 Zu viel Diskussion

🞎 Zu viel Kleingruppenarbeit und Selbstlernen

🞎 Für mich genau richtig

|  | Trifft nicht zu |  | Neutral |  | Trifft genau zu |
| --- | --- | --- | --- | --- | --- |
| 1. Die Kursinhalte entsprachen meinen Erwartungen. | 1 | 2 | 3 | 4 | 5 |
| 1. Der Wissenszuwachs war groß, ich habe viel gelernt. | 1 | 2 | 3 | 4 | 5 |
| 1. Der Problembezug der angebotenen Inhalte war hoch. | 1 | 2 | 3 | 4 | 5 |
| 1. Ich habe im Rahmen der Veranstaltung neue Techniken gelernt. | 1 | 2 | 3 | 4 | 5 |
| 1. Der Kurs war gut strukturiert. | 1 | 2 | 3 | 4 | 5 |
| 1. Der Kurs war so gestaltet, dass ich effizient lernen konnte. | 1 | 2 | 3 | 4 | 5 |
| 1. Ich habe Hilfestellungen für die praktische Durchführung erhalten. | 1 | 2 | 3 | 4 | 5 |
| 1. Ich werde das neu gewonnene Wissen in Zukunft gut anwenden können. | 1 | 2 | 3 | 4 | 5 |
| 1. Die Kursziele wurden erreicht. | 1 | 2 | 3 | 4 | 5 |
|  | Trifft nicht zu |  | Neutral |  | Trifft genau zu |
| 1. Ich wurde als Teilnehmer aktiv eingebunden. | 1 | 2 | 3 | 4 | 5 |
| 1. Der Kurs war lebendig, interessant und praxisbezogen gestaltet. | 1 | 2 | 3 | 4 | 5 |
| 1. Medien (Flipchart, Beamer, Simulatoren, Workstations) wurden adäquat eingesetzt. | 1 | 2 | 3 | 4 | 5 |
| 1. Die Lehrinhalte während der Sitzungen wurden am Ende jeder Einheit gut zusammengefasst. | 1 | 2 | 3 | 4 | 5 |
| 1. Raum für Fragen und Diskussionen wurde ausreichend zur Verfügung gestellt. | 1 | 2 | 3 | 4 | 5 |
| 1. Die Tutoren waren freundlich und gingen auf meine Fragen ein. | 1 | 2 | 3 | 4 | 5 |
| 1. Die wichtigsten Punkte wurden gut erläutert und illustriert. | 1 | 2 | 3 | 4 | 5 |
| 1. Die Leistung der Kursleitung war insgesamt gut. | 1 | 2 | 3 | 4 | 5 |
| 1. Der Kurs hat mir Spaß gemacht. | 1 | 2 | 3 | 4 | 5 |
| 1. Ich werde diesen Kurs weiterempfehlen. | 1 | 2 | 3 | 4 | 5 |
| 1. Ich plane an weiteren TTT-Kursen teilzunehmen. | 1 | 2 | 3 | 4 | 5 |

1. Was hat Ihnen am Kurs am besten gefallen? Welchen Kursteil fanden Sie am effektivsten? (Freitext)

...........................................................................................................................................................

...........................................................................................................................................................

............................................................................................................................................................

............................................................................................................................................................

1. Wie hätte der Kurs effektiver gestaltet werden können? (Freitext)

...........................................................................................................................................................

...........................................................................................................................................................

............................................................................................................................................................

............................................................................................................................................................

**GLOBALE BEWERTUNGEN (Schulnoten 1 – 6)**

1. Der Kurs war insgesamt

🞎 Sehr gut

🞎 Gut

🞎 Befriedigend

🞎 Ausreichend

🞎 Mangelhaft

🞎 Ungenügend

1. Die Kursleitung durch **###** war insgesamt

🞎 Sehr gut

🞎 Gut

🞎 Befriedigend

🞎 Ausreichend

🞎 Mangelhaft

🞎 Ungenügend

**FRAGEBOGEN**

1. ALLGEMEINER TEIL
2. EINGANGSFRAGEBOGEN
3. EVALUATIONSFRAGEBOGEN
4. **ABSCHLUSSFRAGEBOGEN**
5. **ALLGEMEINER TEIL**

**Kurs absolviert in:**

**Datum des Kurses:**

**Alter:**

**Fachdisziplin:**

**Dienstjahre:**

1. **ABSCHLUSSFRAGEBOGEN (POST 2)**
2. Bei welchen Lehrveranstaltungen haben Sie seit dem TTT-Kurs als Dozent mitgewirkt?

🞎 Vorlesung

🞎 Seminar

🞎 Praktikum

🞎 Staatsexamen

🞎 OSCE

🞎 Testate

🞎 Keine

1. Haben Sie seit dem TTT-Kurs weitere medizindidaktische Fortbildungen besucht?

🞎 Ja

Wenn ja, welche? ……………………………………………………………………

🞎 Nein

1. Welche im TTT-Kurs erlernten Methoden haben Sie bislang praktisch umgesetzt? (Mehrfachnennungen möglich)

🞎 Briefing

🞎 Debriefing/Feedback

🞎 4-Step-Approach nach Peyton

🞎 Mentales Training

🞎 Teachable Moments

🞎 MiniCex

🞎 DOPS

1. Haben Sie seit dem TTT-Kurs Ihre Lehrveranstaltungen modifiziert?

🞎 Ja

Wenn ja, wie? ………………………………………………………………………………………

………………………………………………………………………………………

………………………………………………………………………………………

🞎 Nein

1. Welche Aufgaben haben Sie seit dem TTT-Kurs an Ihre Studierenden, nach Einweisung, zur routinemäßigen, selbstständigen Durchführung delegiert? (Mehrfachantworten möglich)

🞎 Blutentnahme

🞎 Bluttransfusion (Bedside Test + Anhängen EK)

🞎 Intravenösen Zugang legen

🞎 Infusionen anhängen

🞎 Anamneseerhebung, Aufnahmegespräch führen

🞎 Körperliche Untersuchung, Aufnahmeuntersuchung durchführen

🞎 Arztbriefschreibung

🞎 Kommunikation (schriftlich, mündlich, persönlich) mit Zuweisern

🞎 Anforderung von Konsilen und/oder Untersuchungen

🞎 Verbandwechsel (komplex; inkl. Débridement, VAC-Verband etc.)

🞎 Pleura-, Aszitespunktion

🞎 Arterielle Punktion, BGA, Arterielle Druckmessung

🞎 Magensonde legen

🞎 Blasenkatheter legen (Einmalkatheter, Dauerkatheter)

🞎 Aufklärungsgespräche für kleinere ambulante Standardeingriffe oder stationäre Diagnostik durchführen

🞎 EKG schreiben

🞎 Analgetika, Antiemetika indizieren und nach Rücksprache verabreichen

🞎 Führen einer Visite

🞎 Port anpunktieren und befahren

🞎 Angehörigengespräche führen

🞎 Codieren

1. Überprüfen Sie die korrekte Durchführung der delegierten Aufgaben und geben Sie Feedback?

🞎 Nie

🞎 Selten

🞎 Gelegentlich

🞎 Häufig

🞎 Immer

1. Nutzen Sie Checklisten zur Bewertung und zum Feedback von praktischen Fertigkeiten?

🞎 Ja

🞎 Nein

| 1. Wenn sich Widerstände auftun, finde ich Mittel und Wege, mich durchzusetzen. | Stimmt nicht | Stimmt kaum | Stimmt eher | Stimmt genau |
| --- | --- | --- | --- | --- |
| 1. Die Lösung schwieriger Probleme gelingt mir immer, wenn ich mich darum bemühe. | Stimmt nicht | Stimmt kaum | Stimmt eher | Stimmt genau |
| 1. In unerwarteten Situationen weiß ich immer, wie ich mich verhalten soll. | Stimmt nicht | Stimmt kaum | Stimmt eher | Stimmt genau |
| 1. Es bereitet mir keine Schwierigkeiten, meine Absichten und Ziele zu verwirklichen. | Stimmt nicht | Stimmt kaum | Stimmt eher | Stimmt genau |
| 1. Auch bei überraschenden Ereignissen glaube ich, dass ich gut mit ihnen zurechtkommen kann. | Stimmt nicht | Stimmt kaum | Stimmt eher | Stimmt genau |
| 1. Schwierigkeiten sehe ich gelassen entgegen, weil ich meinen Fähigkeiten immer vertrauen kann. | Stimmt nicht | Stimmt kaum | Stimmt eher | Stimmt genau |
| 1. Was auch immer passiert, ich werde schon klarkommen. | Stimmt nicht | Stimmt kaum | Stimmt eher | Stimmt genau |
| 1. Für jedes Problem kann ich eine Lösung finden. | Stimmt nicht | Stimmt kaum | Stimmt eher | Stimmt genau |
| 1. Wenn eine neue Sache auf mich zukommt, weiß ich, wie ich damit umgehen kann. | Stimmt nicht | Stimmt kaum | Stimmt eher | Stimmt genau |
| 1. Wenn ein Problem auftaucht, kann ich es aus eigener Kraft meistern. | Stimmt nicht | Stimmt kaum | Stimmt eher | Stimmt genau |

| 1. Ich lehre, weil ich es sehr wichtig finde, meinen Teil dazu beizutragen, dass die Studierenden später gute Ärzte werden. | Stimmt nicht | Stimmt kaum | Keine Meinung | Stimmt eher | Stimmt genau |
| --- | --- | --- | --- | --- | --- |
| 1. Ich lehre, weil ich überzeugt bin, dass es zu den Pflichten eines Arztes gehört, sein Wissen weiterzugeben. | Stimmt nicht | Stimmt kaum | Keine Meinung | Stimmt eher | Stimmt genau |
| 1. Ich lehre vor allem, weil meine Vorgesetzten es von mir erwarten. | Stimmt nicht | Stimmt kaum | Keine Meinung | Stimmt eher | Stimmt genau |
| 1. Ich lehre, obwohl die Lehre mir eher gleichgültig im Vergleich zu meinen anderen beruflichen Tätigkeiten ist. | Stimmt nicht | Stimmt kaum | Keine Meinung | Stimmt eher | Stimmt genau |
| 1. Ich lehre vor allem, weil es zu meinen Arbeitsaufgaben gehört. | Stimmt nicht | Stimmt kaum | Keine Meinung | Stimmt eher | Stimmt genau |
| 1. Ich freue mich meistens auf die nächste Lehreinheit. | Stimmt nicht | Stimmt kaum | Keine Meinung | Stimmt eher | Stimmt genau |
| 1. Ich lehre, obwohl ich fast nie Lust darauf habe. | Stimmt nicht | Stimmt kaum | Keine Meinung | Stimmt eher | Stimmt genau |
| 1. Mein Unterricht macht mir meistens Spaß. | Stimmt nicht | Stimmt kaum | Keine Meinung | Stimmt eher | Stimmt genau |
| 1. Ich lehre, obwohl ich die Lehre oft als lästige Pflicht empfinde. | Stimmt nicht | Stimmt kaum | Keine Meinung | Stimmt eher | Stimmt genau |
| 1. Ich lehre, weil ich sonst ein schlechtes Gefühl meinen Kollegen gegenüber hätte. | Stimmt nicht | Stimmt kaum | Keine Meinung | Stimmt eher | Stimmt genau |
| 1. Während des Unterrichts fühle ich mich ganz in meinem Element. | Stimmt nicht | Stimmt kaum | Keine Meinung | Stimmt eher | Stimmt genau |
| 1. Ich lehre, weil ich den Unterricht benötige, um meine beruflichen Ziele zu erreichen. | Stimmt nicht | Stimmt kaum | Keine Meinung | Stimmt eher | Stimmt genau |
| 1. Ich lehre, weil ich sonst ein schlechtes Gewissen gegenüber meinen Vorgesetzten hätte. | Stimmt nicht | Stimmt kaum | Keine Meinung | Stimmt eher | Stimmt genau |
| 1. Die Lehre stellt eine Bereicherung meines Arbeitsalltags dar. | Stimmt nicht | Stimmt kaum | Keine Meinung | Stimmt eher | Stimmt genau |
| 1. Ich lehre, weil es mich beruflich voranbringt. | Stimmt nicht | Stimmt kaum | Keine Meinung | Stimmt eher | Stimmt genau |
| 1. Ich lehre vor allem, weil ich sonst Schwierigkeiten mit meinen Vorgesetzten bekäme. | Stimmt nicht | Stimmt kaum | Keine Meinung | Stimmt eher | Stimmt genau |
| 1. Ich lehre, weil mich dies in meiner Karriere voranbringen könnte. | Stimmt nicht | Stimmt kaum | Keine Meinung | Stimmt eher | Stimmt genau |
| 1. Ich lehre, weil ich die Inhalte meines Unterrichts wichtig finde. | Stimmt nicht | Stimmt kaum | Keine Meinung | Stimmt eher | Stimmt genau |

| 1. Ich bin sicher, dass ich auch mit problematischen Studierenden in guten Kontakt kommen kann. | Stimmt nicht | Stimmt kaum | Stimmt eher | Stimmt genau |
| --- | --- | --- | --- | --- |
| 1. Ich weiß, dass ich es schaffe, selbst den problematischsten Studierenden den prüfungsrelevanten Stoff zu vermitteln. | Stimmt nicht | Stimmt kaum | Stimmt eher | Stimmt genau |
| 1. Ich bin mir sicher, dass ich mich in Zukunft auf individuelle Probleme der Studierenden besser einstellen kann. | Stimmt nicht | Stimmt kaum | Stimmt eher | Stimmt genau |
| 1. Selbst wenn mein Unterricht gestört wird, bin ich mir sicher, die notwendige Souveränität bewahren zu können. | Stimmt nicht | Stimmt kaum | Stimmt eher | Stimmt genau |
| 1. Selbst wenn es mir mal nicht so gut geht, kann ich doch im Unterricht immer noch gut auf die Studierenden eingehen. | Stimmt nicht | Stimmt kaum | Stimmt eher | Stimmt genau |
| 1. Auch wenn ich mich noch so sehr für die Entwicklung meiner Studierenden engagiere, weiß ich, dass ich nicht viel erreichen kann. | Stimmt nicht | Stimmt kaum | Stimmt eher | Stimmt genau |
| 1. Ich bin mir sicher, dass ich kreative Ideen entwickeln kann, mit denen ich ungünstige Unterrichtsstrukturen verändere. | Stimmt nicht | Stimmt kaum | Stimmt eher | Stimmt genau |
| 1. Ich traue mir zu, die Studierenden für neue Projekte zu begeistern. | Stimmt nicht | Stimmt kaum | Stimmt eher | Stimmt genau |
| 1. Ich kann innovative Veränderungen auch gegenüber skeptischen Kollegen durchsetzen. | Stimmt nicht | Stimmt kaum | Stimmt eher | Stimmt genau |
